# Gait analysis of C57BL/6 mice with complete Freund's adjuvant-induced arthritis using the CatWalk system

**DOI:** 10.1186/1471-2474-14-14

**Published:** 2013-01-08

**Authors:** Subramanian S Parvathy, Willias Masocha

**Affiliations:** 1 Department of Pharmacology and Therapeutics, Faculty of Pharmacy, Kuwait University, P.O. Box 24923, Safat, 13110, Kuwait

**Keywords:** Arthritis, Complete Freund’s adjuvant, Gait analysis, Catwalk, Indomethacin, Static and dynamic gait parameters

## Abstract

**Background:**

The CatWalk system, a video based automated gait analysis system developed to evaluate footfall and gait changes in rodents, has been used for studying rodent models of arthritis, mainly the rat model. However, it has not been used to study static and dynamic gait parameters in mice with Complete Freund’s adjuvant (CFA). CFA is used extensively to induce arthritis in rodents including mice.

**Methods:**

The CatWalk system was used to study gait of freely moving mice with CFA-induced monoarthritis and evaluate pharmacological pain relief in this model of arthritis. CFA (20 μl) was injected intra-articularly into the right hind (RH) limb ankle joint through the Achilles tendon of C57BL/6 mice.

**Results:**

Mice had less regularity in their walking patterns after CFA inoculation compared to baseline walking patterns, which was significant at 2 days post inoculation (dpi). The mice also showed changes in static parameters (paw pressure (light intensity) and print area) as well as dynamic parameters (stance phase duration, swing phase duration and speed, and duty cycle). The ratio of the RH limb (ipslateral) to the left hind (LH) limb (contralateral) for paw pressure, print area, stance phase duration, duty cycle (stance phase duration/sum of stance and swing phase duration), and swing speed were significantly reduced compared to baseline ratios at 1–6 and/or 7 dpi. On the other hand, RH/LH limb ratio of the swing phase duration increased at 3 dpi compared to baseline values. Treatment with indomethacin (10 mg/kg) improved or restored the gait parameters of CFA inoculated mice i.e. similar to baseline values or LH limb.

**Conclusions:**

These data show that the CatWalk system can be used to assess static and dynamic gait changes and pharmacological pain relief in freely moving mice with CFA-induced monoarthritis.

## Background

Gait analysis has been used in patients with rheumatoid arthritis to study both static and dynamic lower limb functional disability or improvement after treatment [1,2]. It has also been used for the study of various models of arthritis [3-6]. The CatWalk system, which is a video based automated gait analysis system was developed to evaluate footfall and gait changes in rodents [7]. It can record both static and dynamic parameters such as paw pressure, print area, stance phase duration, swing phase duration, stride length as well as interlimb coordination. Recently, the CatWalk system has been used for studying rodent models of arthritis, mainly the rat model [8-16]. Functional changes caused by arthritis and the effects of analgesic drugs have been analyzed in some of these models using the CatWalk system [8-10,12,15]. The evaluation of the usability of this system in different mice models of arthritis is important taking into consideration that mice are extensively used in the study of the disease, both in the pathogenesis of the disease and search for therapeutic targets and molecules, and also that mice are the preferred animals for genetic modification to study biology and disease [17]. The background mouse strain most commonly used for the production of transgenic mice is the C57BL/6 (http://jaxmice.jax.org/strain/000664.html). Two previous studies described the use of the CatWalk system in assessment of weight bearing changes and effects of a non-steroidal anti-inflammatory drug in a lipopolysaccharide (LPS)-induced monoarthritic C57BL/6 mouse model [8,15]. However, the analysis was limited to static changes, print area and weight bearing, only. Beside the CatWalk system, other gait analysis systems have been used to evaluate gait in mice with arthritis. One study used DigiGait Imaging System (Mouse Specifics, Inc.), which is to some extent similar to the CatWalk system, to evaluate both static and dynamic parameters in mice with collagen-induced arthritis (CIA) [6]. Locomotion has also been studied in mice with CIA using a computerized tracking system and image analyzer (EnthoVision 3.1, Noldus) and was found to be correlated to clinical arthritis [18].

In the current study the CatWalk system was used to evaluate gait of freely moving C57BL/6 mice with complete Freund’s adjuvant (CFA)-induced monoarthritis and also to evaluate pharmacological pain relief in this model of arthritis. Both static and dynamic parameters were analyzed in arthritic mice taking into consideration that it has been shown in human patients with rheumatoid arthritis that there is close correlation between lower limb pain with dynamic parameters such as speed and stride length [19].

## Methods

### Animals

Female C57BL/6 mice (8 to 12 weeks old; 20 – 30 g, n = 55) were used and were supplied by the breeding unit at the Health Sciences Center, Kuwait University, Kuwait, and were kept in temperature controlled (24 ± 1°C) rooms with food and water *ad libitum*. All experiments were performed during the same period of the day (8:00 AM to 4:00 PM) to exclude diurnal variations in pharmacological effects. The animals were handled in compliance with Animal Resources Centre of the Kuwait University Health Sciences Center guidelines and the European Communities Council Directive 86/609 for the care of laboratory animals and ethical guidelines for research in experimental pain with conscious animals [20]. All procedures were approved by the Animal Resources Centre of the Kuwait University Health Sciences Center.

### Induction of arthritis

Monoarthritis was induced as described previously [21]. Briefly, mice were anaesthetized with isoflurane and 20 μl of complete Freund’s adjuvant (CFA, each ml contained 1 mg of *Mycobacterium tuberculosis* H37Ra, ATCC 25177, heat killed and dried, 0.85 ml mineral oil and 0.15 ml mannide monooleate) (Sigma–Aldrich, St Louis, MO, USA), was injected intra-articularly into the right hind limb ankle joint through the Achilles tendon using a 29½-gauge needle. Phosphate buffered saline (PBS, 20 μl), was administered in the same way to the control group to evaluate the effects of the injection and volume of liquid injected intra-articulary, because CFA has no vehicle to use as a control.

### Gait analysis

Gait parameters of freely moving mice were measured using the Catwalk gait analysis system (Noldus Information Technology, The Netherlands) as described previously [7,15]. Briefly, the CatWalk instrument consists of an enclosed walkway with a glass plate, a fluorescent lamp which emits light inside the glass plate, a high speed colour camera, and a recording and analysis software to assess gait of rodents. Each mouse was placed individually in the CatWalk walkway and allowed to walk freely and traverse from one side to the other of the walkway glass plate. Mice were trained as described previously [15] and gait parameters assessed before (baseline) and after (daily for 7 days) CFA or PBS inoculation. The recordings were carried out when the room was completely dark, except for the light from the computer screen. Where the mouse paws made contact with the glass plate, light was reflected down and the illuminated contact areas recorded with a high speed colour video camera that was positioned underneath the glass plate connected to a computer that runs the CatWalk software 7.1. The software automatically labelled all the areas containing pixels above the set threshold (7 pixels). These areas were identified and assigned to the respective paws. Analysis of the recording generated a wide range of parameters of which only the following were analysed:

I. Interlimb coordination: measured as regularity index, which is the percentage of normal step sequences,

II. Paw pressure: light intensity, which is the mean brightness of all pixels of the print at maximum paw contact, ranging from 0–255 arbitrary units,

III. Paw print area: complete surface area contacted by the paw during a stance phase,

IV. Stance phase: duration in seconds of contact of a paw with the glass plate in a step cycle i.e. stance phase + swing phase,

V. Swing phase: duration in seconds of no contact of a paw with the glass plate in a step cycle,

VI. Duty cycle: stance duration as a percentage of the step cycle duration i.e. stance phase/(stance + swing phases) × 100

VII. Stride length: distance between successive placements of the same paw

VIII. Swing speed: speed of a paw during swing phase i.e. stride length/swing phase duration

### Drug treatment

Mice were treated intraperitoneally with indomethacin 0.1 (n = 8), 1 (n = 9) and 10 (n = 9) mg/kg (Sigma–Aldrich, St Louis, MO, USA) or its vehicle (peanut oil, Sigma-Aldrich, USA, n = 10), 48 hours after CFA inoculation based on the gait analysis results (see Results section). The mice were then made to cross the Catwalk walkway at 1, 2 and 3 h post-indomethacin treatment.

### Data analysis

Statistical analyses were performed using repeated measures two-way analysis of variance (ANOVA) followed by Bonferroni post-tests or one-way ANOVA followed by Dunnett's multiple comparison test. The differences were considered significant at p < 0.05. The results in the text and figures are expressed as the means ± S.E.M.

## Results

### Interlimb coordination

**Figure 1:**
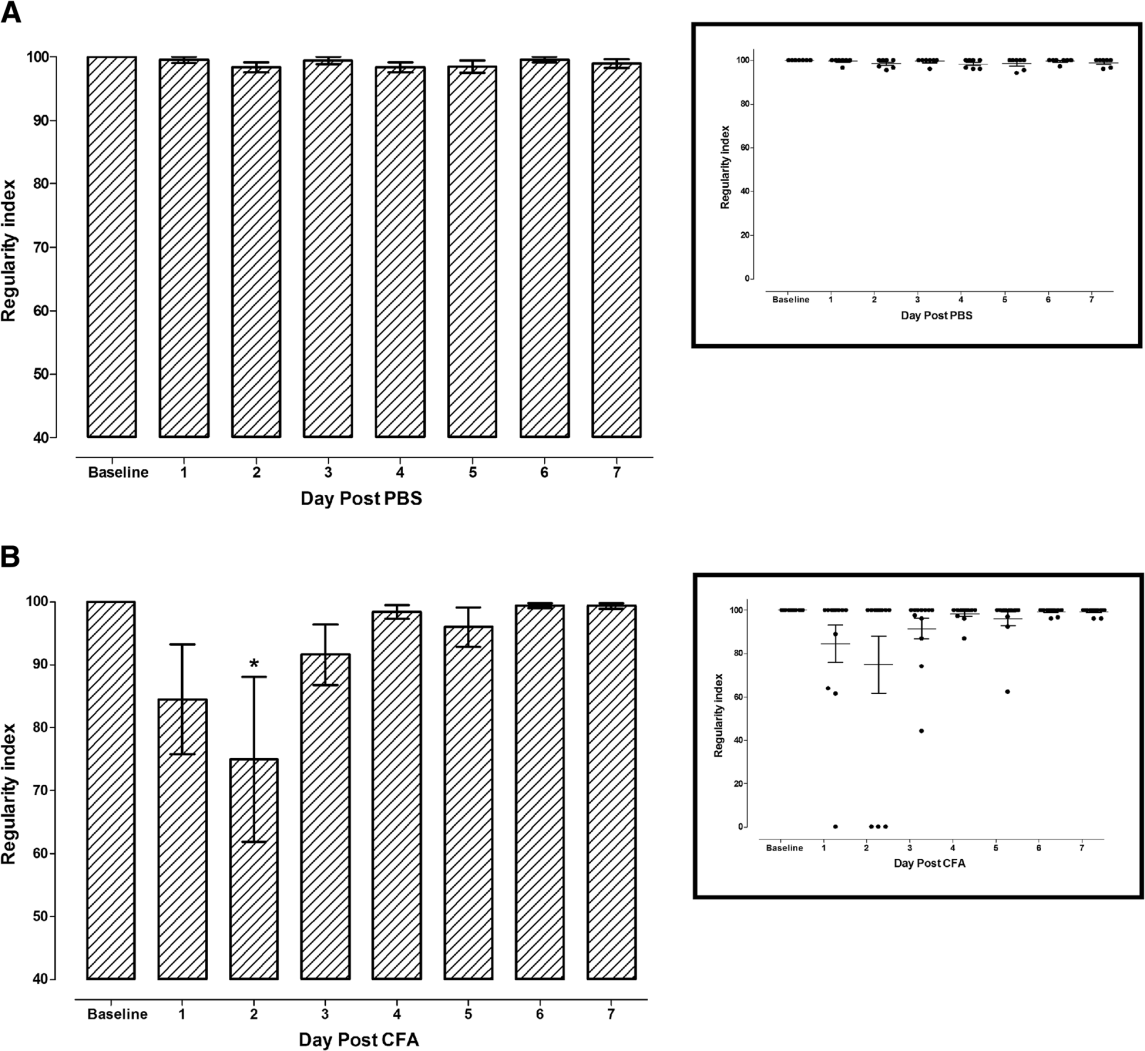
**Time course of regularity index before (baseline) and after inoculation of (A) phosphate buffered saline (PBS) or (B) complete Freund’s adjuvant (CFA) in the right hind limb.** Each point represents the mean ± S.E.M of the values obtained from 7 (PBS) to 12 (CFA) animals. Statistically significant differences in comparison with baseline: ^*^ p < 0.05 (one-way ANOVA followed by Dunnett's Multiple Comparison Test).

The baseline inter-limb coordination of the mice measured by regularity index was 100% (Figure 1), indicating that the paw placements of all mice followed a normal step sequence. The regularity index did not change after intra-articular inoculation of PBS in the right hind (RH) limb (Figure 1A). However, the regularity index dropped after intra-articular inoculation of CFA in the RH limb reaching statistical significance only at 2 days post CFA inoculation (dpi) (p < 0.05; Figure 1B). This significant drop in regularity index at 2 dpi was mostly due to some mice that completely avoided stepping with the RH limb (about 25% of the mice), thus were walking with three limbs instead of four resulting in a zero regularity index for these mice (Figure 1B scatter dot plot).

### Paw pressure and print area

**Figure 2:**
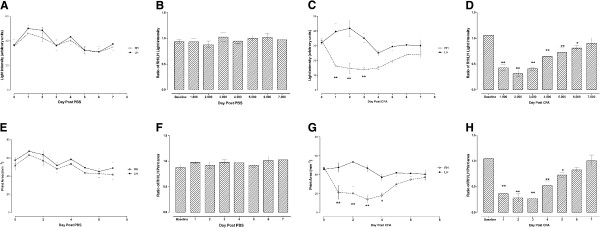
**Time course of static gait parameters in mice with (A, B, E and F) phosphate buffered saline (PBS) or (C, D, G and H) complete Freund’s adjuvant (CFA)-injected intra-articularly in the right hind (RH) limb i.e. paw pressure measured as light intensity (arbitrary units) (A and C) and ratio of light intensity between RH and left hind(LH) limbs (B and D), print area (E *and *G) and ratio of print area between RH and LH limbs (F and H).** Each point represents the mean ± S.E.M of the values obtained from 7 (PBS) to 12 (CFA) animals. Statistically significant differences between RH and LH at the same time point post-CFA administration (**C** and **G**) * p < 0.05 and. ^**^ p < 0.01 (repeated measures two-way ANOVA followed by Bonferroni post-tests) and in comparison with ratio of RH/LH paw pressure or print area before CFA injection (**D** and **H**): ^*^ p < 0.05 and ^**^ p < 0.01(one-way ANOVA followed by Dunnett's Multiple Comparison Test).

The paw pressure (light intensity) of the RH limb of PBS-inoculated mice did not change for 7 dpi compared to the left hind (LH) limb (Figure 2A). Weight bearing changes were calculated using the ratio of RH to the LH limb paw pressure as described previously [8] and [15]. The RH/LH paw pressure ratio of PBS-inoculated mice also did not change (Figure 2B). On the other hand, the paw pressure of the RH limb of CFA-inoculated mice decreased significantly from 1 to 3 dpi compared to the LH limb (Figure 2C), and the RH/LH paw pressure ratio decreased significantly from 1 to 6 dpi compared to baseline values (Figure 2D).

The paw print area of the RH limb and the RH/LH paw print area ratio of PBS-inoculated mice did not change for 7 dpi compared to the LH limb or baseline, respectively (Figure 2E and F) The paw print area of the RH limb of CFA-inoculated mice decreased significantly from 1 to 4 dpi compared to the LH limb (Figure 2G); similarly the RH/LH paw print area ratio decreased significantly from 1 to 5 dpi compared to baseline values (Figure 2H).

### Dynamic paw parameters

**Figure 3:**
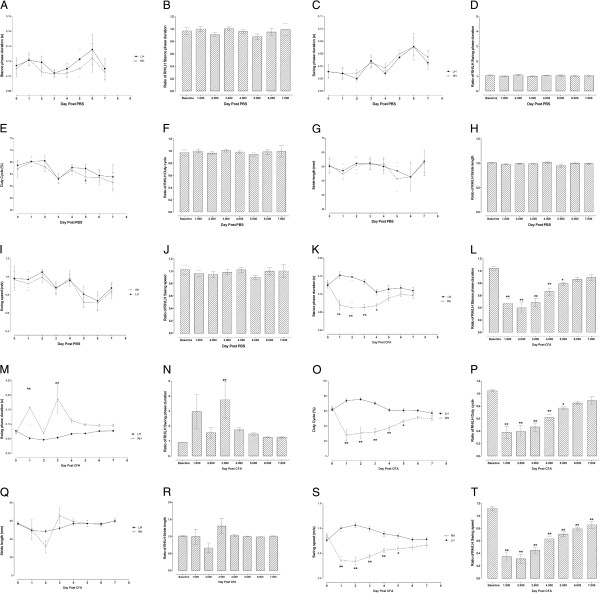
**Time course of dynamic gait parameters (A-J) in mice with phosphate buffered saline (PBS) or (K-T) complete Freund’s adjuvant (CFA)-injected intra-articularly in the right hind (RH) limb i.e. stance phase duration (A and K) and ratio of stance phase duration between RH and left hind (LH) limbs (B and L), swing phase duration (C and M) and ratio of swing phase duration between RH and LH limbs (D and N), duty cycle {stance phase duration/(stance + swing phases duration) x 100} (E and O) and ratio of duty cycle between RH and LH limbs (F and P), stride length (G and Q) and ratio of stride length between RH and LH limbs (H and R), swing speed (I and S) and ratio of swing speed between RH and LH limbs (J and T).** Each point represents the mean ± S.E.M of the values obtained from 7 (PBS) to 12 (CFA) animals. Statistically significant differences between RH and LH at the same time point post-CFA administration (**K, M, O** and **S**). ^*^ p < 0.05 and ^**^ p < 0.01 (repeated measures two-way ANOVA followed by Bonferroni post-tests) and in comparison with ratio of RH/LH values before CFA injection (**L, N, P** and **T**): ^*^ p < 0.05 and ^**^ p < 0.01 (one-way ANOVA followed by Dunnett's Multiple Comparison Test).

All the dynamic parameters evaluated (duration of the stance phase; duration of the swing phase; duty cycle, which represents stance duration as a percentage of step cycle duration; stride length and swing speed) of PBS-inoculated mice did not change for the RH limb or the RH/LH limb ratio for 7 dpi compared to the LH limb or baseline, respectively (Figure 3A-J).

The duration of the stance phase of the RH limb of CFA-inoculated mice decreased significantly from 1 to 4 dpi compared to the LH limb (Figure 3K). The RH/LH stance phase duration ratio decreased significantly from 1 to 5 dpi compared to baseline values (Figure 3L). On the other hand, the duration of the swing phase of the RH limb of CFA-inoculated mice increased significantly at 1 and 3 dpi compared to the LH limb (Figure 3M). The RH/LH swing phase duration ratio increased significantly at 3 dpi compared to baseline values (Figure 3N). The duty cycle of the RH limb of CFA-inoculated mice decreased significantly from 1 to 5 dpi compared to the LH limb (Figure 3O). The RH/LH duty cycle ratio decreased significantly from 1 to 5 dpi compared to baseline values (Figure 3P), similar to the stance phase duration. The stride length of the RH limb of CFA-inoculated mice did not change significantly for 7 dpi compared to the LH limb (p > 0.05; Figure 3Q). The RH/LH stride length ratio also did not change significantly after CFA inoculation compared to baseline values (p > 0.05; Figure 3R). The swing speed of the RH limb of CFA-inoculated mice decreased significantly from 1 to 5 dpi compared to the LH limb (Figure 3S). The RH/LH swing speed ratio decreased significantly from 1 to 7 dpi (longer than all other parameters) compared to baseline values (Figure 3T).

### Effects of treatment with indomethacin on gait parameters of mice with CFA-induced monoarthritis

The effects of treatment of mice with CFA-induced monoarthritis with different doses indomethacin (0.1 - 10 mg/kg) on the gait changes were evaluated at 48 hours post CFA-inoculation. Indomethacin at a dose of 0.1 mg/kg did not affect any of the gait changes in mice with CFA-induced monoarthritis (data not shown).

**Figure 4:**
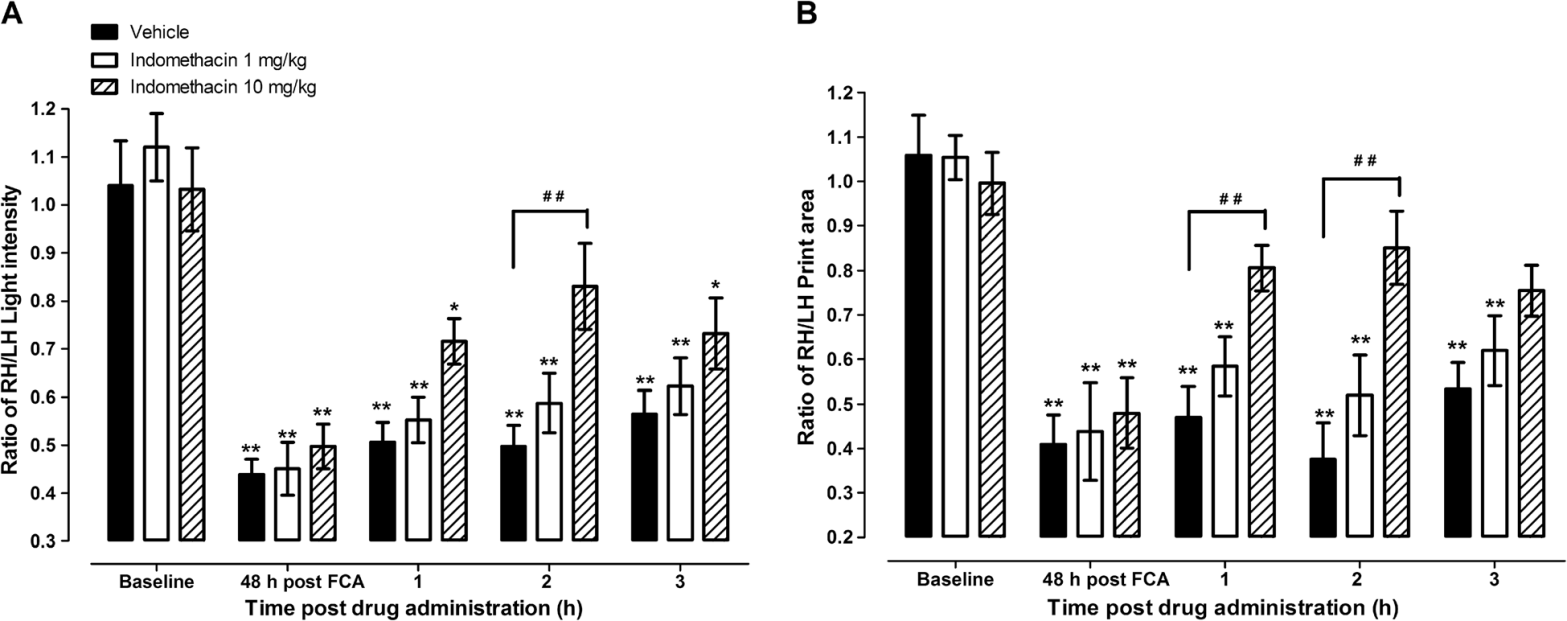
**Effects of indomethacin 1 and 10 mg/kg on (A) weight bearing (measured as ratio of light intensity between right hind (RH) and left hind (LH) limbs and (B) ratio of RH/LH print area of mice with complete Freund’s adjuvant (CFA)-induced arthritis.** The drugs or their vehicles were administered at 48 hours post-CFA administration and their effects measured at 1, 2 and 3 hours after drug treatment. Each point represents the mean ± S.E.M of the values obtained from 9 to 10 animals. Statistically significant differences in comparison with baseline values: * p < 0.05 and ** p < 0.01 (one-way ANOVA followed by Dunnett's Multiple Comparison Test) and in comparison with drug vehicle treated group at the same time point post treatment: ^#^ p < 0.05 and ^# #^ p < 0.01 (repeated measures two-way ANOVA followed by Bonferroni post-tests).

The RH/LH ratios of the static parameters paw pressure and print area were significantly reduced at 48 h after CFA inoculation (2 dpi), similar to the data above (p < 0.01; Figure 4). Treatment of monoarthritic mice with indomethacin 10 mg/kg, but not 1 mg/kg, alleviated the paw pressure and print area RH/LH ratio deficits at 1, 2 and 3 hours post drug treatment (p > 0.05 between baseline values and monoarthritic treated mice). The effect of the drug was most significant at 2 h post-treatment i.e. monoarthritic mice treated with indomethacin 10 mg/kg had significantly higher RH/LH ratios compared to vehicle-treated monoarthritic mice (Figure 4).

**Figure 5:**
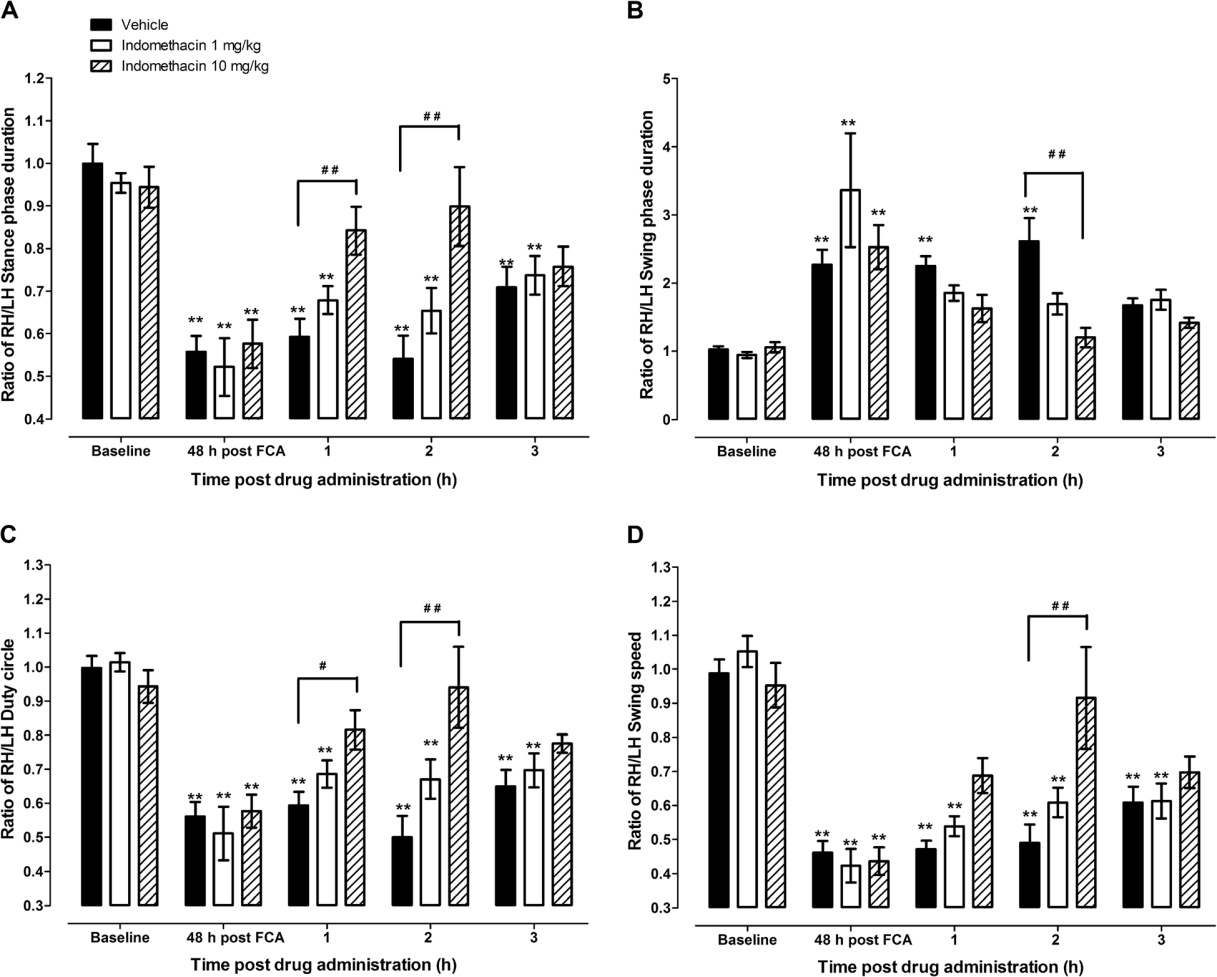
**Effects of indomethacin 1 and 10 mg/kg on (A) ratio of stance phase duration between right hind (RH) and left hind (LH) limbs, (B) ratio of RH/LH swing phase duration, (C) ratio of RH/LH duty cycle {stance phase duration/(stance + swing phases duration) x 100}, and (D) ratio of RH/LH swing speed duration of mice with complete Freund’s adjuvant (CFA)-induced arthritis.** The drugs or their vehicles were administered at 48 hours post-CFA administration and their effects measured at 1, 2 and 3 hours after drug treatment. Each point represents the mean ± S.E.M of the values obtained from 9 to 10 animals. Statistically significant differences in comparison with baseline values: ** p < 0.01 (one-way ANOVA followed by Dunnett's Multiple Comparison Test) and in comparison with drug vehicle treated group at the same time point post treatment: ^# #^ p < 0.01 (repeated measures two-way ANOVA followed by Bonferroni post-tests).

The RH/LH ratios of the dynamic parameters (stance phase duration, swing phase duration and speed, and duty cycle) were significantly changed at 48 h after CFA inoculation i.e. stance phase duration, swing speed, and duty cycle decreased, whereas swing phase duration increased (p < 0.01; Figure 5). Treatment of monoarthritic mice with indomethacin 10 mg/kg alleviated the stance phase duration, swing phase duration and speed, and duty cycle RH/LH ratio deficits at 1, 2 and 3 hours post drug treatment (p > 0.05 between baseline values and monoarthritic treated mice). The effect of the drug was most significant at 2 h post-treatment i.e. monoarthritic mice treated with indomethacin 10 mg/kg had significantly higher RH/LH ratios compared to vehicle-treated monoarthtic mice (Figure 5). The swing phase duration was more sensitive to the effects of treatment with indomethacin since it was the only parameter alleviated by a lower dose of indomethacin (p > 0.05 between baseline values and monoarthritic treated mice; Figure 5).

## Discussion

The results of this study show that CFA-induced monoarthritis in C57BL/6 mice results in changes in static and dynamic gait parameters which can be quantified by the CatWalk system. These changes are not due to the effects of the injection process or volume of liquid because intra-articular administration of PBS did not affect any of the mice gait parameters. The extent of alleviation of the CFA-induced monoarthritis deficits in gait parameters by treatment with a non-steroidal anti-inflammatory drug, indomethacin, can also be quantified using the CatWalk system.

Using a systematic literature search within the PubMed database we found nine studies that have used the CatWalk system to study rodent models of arthritis [8-16]. However, almost all of these studies (seven out of nine) have been dedicated to the study of rat models of arthritis [9-14,16]. The two studies that used mice used the C57BL/6 mice, inoculated with LPS into the RH limb, evaluated principally static parameters i.e. paw pressure and print area [8,15]. However, other gait analysis systems such as the DigiGait Imaging System (Mouse Specifics, Inc.), have been used to evaluate both static and dynamic parameters in mice with CIA [6].

Complete Freund’s adjuvant has been used extensively to induce arthritis in rodents including mice [21-23]. Using an observer based-rating scale, mice with CFA-induced monathritis have been reported to have impaired stance and gait [21]. Recently, a study using rats showed that CFA-induced monoarthritis resulted in deficits in load/weight bearing in the ipslateral limb [16]. In the current study, using the automated CatWalk system, reduced weight bearing on the ipslateral limb in mice with CFA-induced monoarthritis was also recorded, which resulted in a reduced weight bearing ratio of the ipslateral to the contralateral hind limb. Akin to the LPS-induced monoarthritis mouse model [15], reduced paw print area was observed in the ipslateral limb of mice with CFA-induced monoarthritis. These deficits were alleviated by treatment with indomethacin, comparable to what was previously observed in the LPS-induced monoarthritis mouse model [15]. These observations resemble the clinical situation in patients taking into consideration that in patients with unilateral knee osteoarthritis pain relief in the affected knee resulted in even load distribution between the legs [24].

Besides weight bearing, dynamic parameters such as velocity and stride length are altered in human patients with rheumatoid arthritis and these changes correlate closely with lower limb pain [19]. This is the first study where changes in dynamic parameters such as stance phase duration, swing phase duration, duty cycle and swing speed quantified by the CatWalk system have been reported in a mouse model of arthritis. Similar to what has been reported in rat models of arthritis stance phase duration and duty cycle (expressed in these other studies as fraction of total step duration or duty factor) [9,13] were decreased in the ipslateral limb compared to the contralateral limb in mice with monoarthritis. Treatment with indomethacin alleviated the deficits in stance phase duration and duty cycle in arthritic mice. Analgesics such as morphine and rofecoxib have also been shown to improve changes related to stance phase duration in rats with carrageenan-induced arthritis [9]. Swing phase duration and speed were also changed in mice with arthritis i.e. swing phase duration was increased and swing speed was decreased. These parameters were improved in mice treated with indomethacin. These findings concur with what has been reported in rats with monoiodoacetate-induced arthritis both in terms of deficits and improvement after treatment with an analgesic, celecoxib [10].

## Conclusion

This mouse model of arthritis induced by intraarticular injection of CFA has weight bearing and temporal gait deficits comparable to other models of arthritis, either rats or mice models. The use of an automated gait analysis system, CatWalk system, in evaluating gait deficits in mice with CFA-induced arthritis can produce quantifiable and easy-to- reproduce data with less observer bias. More importantly, these deficits and their alleviation by drug treatment correspond to what is observed in some human patients with arthritis.

## Abbreviations

ANOVA: Analysis of variance; CFA: Complete Freund’s adjuvant; CNS: Central nervous system; i.p: Intraperitoneally; LH: Left hind limb; LPS: Lipopolysaccharide; PBS: Phosphate buffered saline; RH: Right hind limb; S.E.M: Error of the mean.

## Competing interests

The authors have no competing interests.

## Authors’ contributions

SSP participated in the acquisition and analysis of data, and helped to edit the manuscript. WM participated in the design of the study, acquisition and analysis of data, and drafting and preparation the final manuscript. Both authors read and approved the final manuscript.

## Pre-publication history

The pre-publication history for this paper can be accessed here:

http://www.biomedcentral.com/1471-2474/14/14/prepub
